# Paper-based microfluidics for DNA diagnostics of malaria in low resource underserved rural communities

**DOI:** 10.1073/pnas.1812296116

**Published:** 2019-02-19

**Authors:** Julien Reboud, Gaolian Xu, Alice Garrett, Moses Adriko, Zhugen Yang, Edridah M. Tukahebwa, Candia Rowell, Jonathan M. Cooper

**Affiliations:** ^a^Division of Biomedical Engineering, University of Glasgow, G12 8LT Glasgow, United Kingdom;; ^b^Nano Biomedical Research Centre, School of Biomedical Engineering, Shanghai Jiao Tong University, 200030 Shanghai, People’s Republic of China;; ^c^Vector Control Division, Ministry of Health, Kampala, Uganda

**Keywords:** malaria, nucleic acid-based tests, paper microfluidics, low-resource settings, point-of-care diagnostics

## Abstract

Rapid, low-cost, species-specific diagnosis, based upon DNA testing, is becoming important in the treatment of patients with infectious diseases. Here, we demonstrate an innovation that uses origami to enable multiplexed, sensitive assays that rival polymerase chain reactions (PCR) laboratory assays and provide high-quality, fast precision diagnostics for malaria. The paper-based microfluidic technology proposed here combines vertical flow sample-processing steps, including paper folding for whole-blood sample preparation, with an isothermal amplification and a lateral flow detection, incorporating a simple visualization system. Studies were performed in village schools in Uganda with individual diagnoses being completed in <50 min (faster than the standard laboratory-based PCR). The tests, which enabled the diagnosis of malaria species in patients from a finger prick of whole blood, were both highly sensitive and specific, detecting malaria in 98% of infected individuals in a double-blind first-in-human study. Our method was more sensitive than other field-based, benchmark techniques, including optical microscopy and industry standard rapid immunodiagnostic tests, both performed by experienced local healthcare teams (which detected malaria in 86% and 83% of cases, respectively). All assays were independently validated using a real-time double-blinded reference PCR assay. We not only demonstrate that advanced, low-cost DNA-based sensors can be implemented in underserved communities at the point of need but also highlight the challenges associated with developing and implementing new diagnostic technologies in the field, without access to laboratories or infrastructure.

## Significance

Populations living in remote rural communities would benefit from rapid, highly sensitive molecular, DNA-based diagnostics to inform the correct and timely treatment of infectious diseases. Such information is also becoming increasingly relevant in global efforts for disease elimination, where the testing of asymptomatic patients is now seen as being important for the identification of disease reservoirs. However, healthcare workers face practical and logistical problems in the implementation of such tests, which often involve complex instrumentation and centralized laboratories. Here we describe innovations in paper microfluidics that enable low-cost, multiplexed DNA-based diagnostics for malaria, delivered, in a first-in-human study, in schools in rural Uganda.

Malaria is a leading cause of morbidity and mortality worldwide. There are >219 million cases in 90 countries, with more than 435,000 deaths in 2017 (1). Despite efforts in recent years surrounding preventive interventions, including bed netting, insecticide spraying, and mass prophylactic drug administration, progress has stalled. The 2018 World Health Organization (WHO) malaria report (1) highlights that, after almost two decades of decline, malaria cases have significantly risen in 13 countries, stating that no significant progress has been made in reducing global malaria between 2015 and 2017 (before then, the number of people contracting the disease had been falling). This has prompted concern that more is needed to combat the epidemic, and the report highlights the need for species-specific diagnostics before treatment. Health workers are increasingly calling for low-cost, rapid diagnostic devices that require no refrigeration, laboratory equipment, or special training, but that can detect parasites even when they are not abundant.

It has also recently become clear that existing standard field tests for many infections, including malaria, are not reliable, and new strategies are now needed to deliver effective diagnostics to reduce the burden of disease. As part of such a strategy, effective, sensitive and accurate, species-specific detection is needed to direct therapy in a more informed manner (2). The importance of identifying disease reservoirs in asymptomatic patients is also particularly important in disease elimination programs and provides a challenge that current techniques cannot meet.

Rural, remote, underserved communities, where many infectious diseases are most prevalent, can bear the largest social, healthcare, and economic burdens. Treatments are often prescribed based on presumptive diagnosis, due to the inability to perform reliable, precise tests on site (3). Light microscopy has long been the “go-to” standard technique used to diagnose malaria in such areas, and requires only a microscope and a trained technician to achieve detection sensitivities of 100 parasites per microliter of blood (4). However, this diagnostic technique cannot detect asymptomatic patients, who have very low, persistent parasite loads in their blood. This is a key challenge, for the identification of these patients is required to treat all infectious reservoirs, thereby working toward important WHO targets concerning disease elimination (5).

When microscopy-based diagnosis is performed by highly trained technicians, it can, however, identify the parasitic species concerned, which is essential for targeting treatment effectively (6). Similarly, point-of-care immunoassays, also known under the generic term of rapid diagnostic tests (RDTs), are based upon a hand-held lateral flow technology and are also widely used in low-resource areas. They are able to detect the presence of species-specific antigens derived from the malarial parasites, from a finger prick of blood in ∼15 min. The methods use no instrumentation and no electrical power, as the blood sample flows through the paper via capillary forces (7). However, the performance of such assays has been limited by their generation of false-positive results, which occur when nonspecific biomolecules present in the blood, such as the rheumatoid factor, for example, react with the test antigens (8). Technical issues linked to capillary flow and to reagent stability in challenging environmental conditions, including high humidity and temperature (9), have also affected the reliability of RDT immunodiagnostics. Test sensitivities are often only 70 to 75% in the field (10), despite being much higher in well-controlled laboratory conditions (11). Their performance is significantly less than globally accepted targets set by key stakeholders, such as the WHO and Foundation for Innovative Diagnostics (FIND), who target 97% sensitivity as a minimal requirement (12). To have a significant impact in diagnosing malaria, in future, RDTs will need to demonstrate improved sensitivity and accuracy, as well as adequate performance under adverse field conditions (13).

Nucleic acid amplification-based tests (NAATs) provide a promising approach for DNA-based malaria diagnostics. These tests both amplify and detect the genomic material of the parasite directly from a patient sample, providing a sensitive, species-specific test that will also identify whether an infection is current rather than historical (6, 14). In addition, the signal used for detection does not depend on the patients’ immune responses, and the technique can be both quantitative and more accurate than immuno-RDTs (15). Such tests are also now seen as being critical to elimination programs, not only for malaria but for other infectious diseases, given that they can detect low levels of asymptomatic infections, which may act as reservoirs for future disease emergence.

In many reference laboratories, PCR-based amplification assays remain the gold-standard NAAT (16), although the requirement for trained staff and external power has limited their application in areas with reduced resources. Loop-mediated isothermal amplification (LAMP) of DNA (17) and other similar isothermal amplification methods have recently emerged as easy-to-use alternatives to PCR (14), owing to greatly simplified hardware requirements. For example, no temperature cycling is required by these techniques, and their amplification products can be detected visually (18). Where power supplies and laboratory infrastructure can be established (such as during an epidemic), NAATs can often be implemented (19, 20). However, these methods have limited multiplexing ability, due to the complex sample preparation steps required (21).

Recently, lateral flow techniques have been combined with PCR for end point detection (22) by using conventional laboratory equipment. The testing of clinical samples for nucleic acids has also been performed for infectious disease diagnosis in resource-limited environments (21, 23), for example, to detect Ebola virus from extracted RNA. For the first time, this has brought NAAT-based diagnostics closer to patients in rural communities, with samples being stored and assayed in a field laboratory facility (24).

We recently reported a diagnostic platform that uses paper folding (origami) to integrate the different blood sample preparation steps that are required for LAMP onto a paper microfluidic device (25). We used this technique in a hospital setting to test clinical samples and demonstrated the identification of specific malarial species in a multiplexed, sample-to-answer, paper-based microfluidic device. This platform was subsequently used successfully in a field-based laboratory setting in India to detect sexually transmitted diseases in cattle (26). However, in both cases, the detection step was based on a fluorescent readout, which presented challenges, both in its performance and its ease of use. For example, the readout was difficult to assess quantitatively in varying levels of ambient light. This optical detection method was particularly ambiguous at low levels of infections, when the signal-to-background was small.

**Fig. 1.**
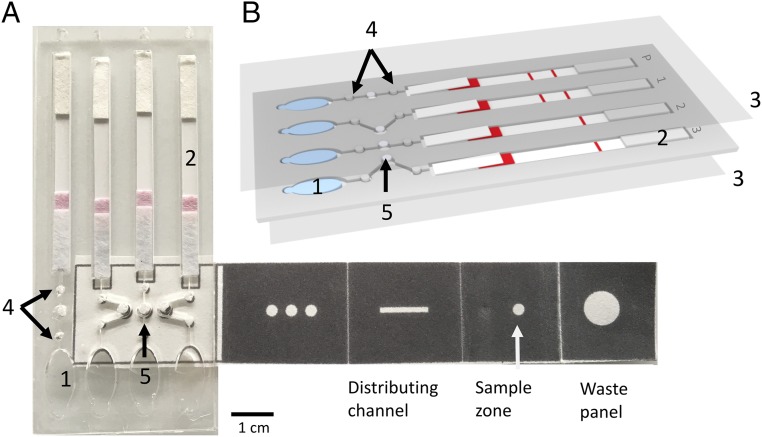
Paper-based microfluidic device that enables multiplex LAMP-based detection of malaria in blood. (*A*) An assembled paper−plastic lateral flow diagnostic device, used for amplification and detection. Attached to this device is a foldable paper strip that is used for DNA extraction and processing. (Fig. 2 shows, in detail, how this strip is folded). The unfolded paper strip consists of dark regions that are created by printing the paper with hydrophobic wax and unpatterned regions that direct sample flow. The rightmost fold is the waste panel, the sample panel to its left shows where the sample is loaded, and the distributing channel is where DNA is eluted for LAMP-based DNA testing. The final (leftmost) fold sits on top of the three LAMP testing chambers (denoted by arrow 5). Pink shading on the lateral flow strip is due to the colored detection particles stored at this position (*SI Appendix*, Fig. S1). The scale bar relates to the whole device. (*B*) A schematic of the plastic microfluidic device, highlighting its key features (1 to 5), namely: 1, buffer chambers, which produce fluid flow, when pressed manually [as a finger pump (29)]; 2, lateral flow DNA detection strip; 3, acetate films to prevent sample evaporation and to enable safe handling; 4, filter paper-based valves, which prevent the LAMP reaction from mixing with the buffer held in the buffer chambers (feature 1) during amplification, while also preventing it from flowing into the lateral flow strip prematurely [the contents of the LAMP reaction volume (feature 5) should only reach the lateral flow strips after amplification, enabled by the flow of buffer from the buffer chambers (feature 1), following manual pumping of the chambers]; and 5, filter paper for the LAMP reaction. The P channel provides a positive control for the LAMP reaction (to control for environmental conditions and handling) using a synthetic DNA target. Red shading bands denote LAMP testing results (*SI Appendix*, Fig. S1).

To overcome this uncertainty in visual signal measurement, we now present a format for a paper-based microfluidic device that uniquely combines our previously published vertical sample-processing steps of the origami device (25) with a microfluidic lateral flow LAMP amplification and detection platform. This facilitates data analysis by healthcare workers, providing an easy-to-use display of the results. Importantly, the integration of this lateral flow readout avoids the inherent ambiguity of fluorescence outputs, supporting the deployment of these diagnostic devices in rural communities (where there is a requirement that results can be readily and accurately interpreted; Fig. 1).

The performance of the device was evaluated in humans in primary schools in St. Kizito and Mayuge Districts, Uganda, on 67 patients. Its effectiveness was benchmarked against two standard field-based techniques, RDT and light microscopy run at the same time, as well as against a laboratory-based, real-time PCR assay, carried out retrospectively.

The sample-processing steps performed by this paper-based microfluidic device included DNA extraction, LAMP isothermal amplification, and lateral flow antibody-based detection of DNA amplicons. The nucleic acid amplification step was demonstrated in the laboratory to characterize the device performance, including 20 negative patient samples (*SI Appendix*, Fig. S2) and, importantly, 67 patients in the field, with amplification being performed either on a simple hot plate (*n* = 59) or in a water bath, maintained at a constant temperature on a cooking stove (*n* = 8).

The test was designed as a multiplexed assay to identify *Plasmodium falciparum*, the endemic malaria-causing parasitic species in the region of study, as well as *Plasmodium* pan (to detect all other parasitic species that cause malaria, which might be present for various reasons, such as human migration). The innovations that we describe demonstrate the potential for such tests, either in the precise diagnosis of disease enabling informed species-specific therapy or, in the future, for global surveillance or screening. The technique is now being evaluated by healthcare professionals from the Uganda Vector Control Division, Ministry of Health, for use in rural areas without access to centralized facilities.

## Results

The paper-based vertical flow origami sample-processing unit and the lateral flow assay, together with LAMP assays and gold-standard (laboratory test) PCR assays, were all developed and optimized for fieldwork, before departure, at the University of Glasgow. The combination of using the origami sample processing combined with a lateral flow assay represents a substantial improvement on our previous work with key advantages over previous studies (25, 26), which had involved reading the results as a fluorescence signal with a flashlight. These devices suffered from difficulties in differentiating the nature and intensity of the fluorescence, particularly in variable ambient light conditions found within rural settings. The incorporation of a semiquantitative lateral flow assay not only speeded up the measurements but also gave increased confidence in the diagnosis.

### Device Design and Fabrication.

**Fig. 2.**
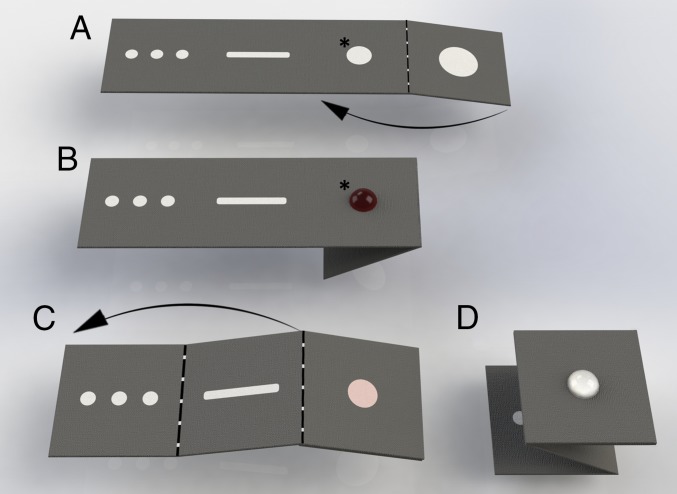
Paper-folding steps for fluidic manipulation. Schematics of how the paper strip in the microfluidic device is folded for each step. The arrows indicate the direction of folding. (*A*) The layout of the multiplex paper device, which is linked to a plastic casing (Fig. 1) that contains the lateral flow DNA detection strip. (*B*) The magnetic beads and the DNA in the sample are mixed and added to the sample zone (*). Impurities are washed off using washing buffer and drained onto the waste pad by folding the paper strip, as shown in *A* (arrow). (*C*) The second (sample zone) and third panels are then folded to come into contact with each other, forming a distributing channel for DNA elution. (*D*). The distributing channel is then folded onto the remaining square, which is then placed against the plastic device to cover the three LAMP chambers (see arrow 5 in Fig. 1); these chambers each contain a 3-mm filter paper frit that receives the DNA into the plastic device. The reagents for the LAMP reaction are then added to the reaction chambers. Movie S1 shows these sample-processing steps using mock samples.

The paper origami device for DNA extraction was fabricated using an inexpensive hot wax printing method (Fig. 1*A*), with adaptations to published methods (25, 27). The folding-based, sample-processing steps are illustrated in Figs. 1 and 2 (and are also detailed in Movie S1). The lateral flow component of the assay was designed in the University of Glasgow and was fabricated by Ustar (28).

A finger-prick blood sample was added to an RDT, to an Eppendorf tube, to a “fast transient analysis” (FTA) card (for retrospective analysis with PCR amplification assays), and to glass slides for thick smears using Giemsa staining and optical microscopy. The same blood was used immediately (before clotting, <2 min) for the microfluidic paper test. Ten microliters of lysis buffer containing magnetic beads was added to 5 µL of blood. After 5 min, a further aliquot of binding buffer was added, and the DNA was captured on the beads, which were transferred to the sample zone of the folded (origami) paper device (Figs. 1*A* and 2*B*) as a 40-µL volume. All residues were then washed off manually by dispensing a volume-independent aliquot of a washing buffer onto the sample reception area; excess buffer and residues were then drained away under capillary force by folding the reception panel against a waste panel (Figs. 1*A* and 2*B*).

The bead-bound DNA was retained within the paper matrix, which was then refolded against distributing channels (Fig. 2 *C* and *D*) before elution buffer was added. Within the distribution channel, the sample was split into three spots (as a multiplex), which themselves then formed the independent LAMP chambers (Fig. 1*A*, arrow 5). The sample-preparation processing requires two manipulations, namely, the addition of washing and elution buffers, a procedure easily performed by local healthcare workers.

In two of the chambers, DNA from *Plasmodium* pan and *P. falciparum* were tested, while a separate chamber was used as a control. The device was sealed with an acetate film to prevent evaporation during the LAMP reaction at 63 °C for 45 min on a hot plate, or in a water bath, heated on a cooking stove. If *Plasmodium* DNA is present in the sample, a specific LAMP reaction takes place in which amplicons are generated with both biotin- and FITC-labeled primers, as a complex bearing two ligands (see *SI Appendix*, Fig. S1, which illustrates how the lateral flow assay works and how its results are visualized).

Following the LAMP reaction, the test sample was then transferred to the lateral flow strips (Fig. 1, location 2) using a finger pump (29). The buffer moves the products of the LAMP reaction laterally into the detection strips, and the assay proceeds under capillary flow. In this way, the vertical sample-processing steps of the origami device were integrated into a lateral flow-mediated amplification and visual readout system. The detection strips contain anti-FITC antibodies and immobilized streptavidin in the test and control lines, respectively. This detection step takes less than 1 min, and results were analyzed by eye (*SI Appendix*, Fig. S1). When the complex, generated by the LAMP assay flows through the lateral flow detection device, the species-specific ligands are captured to generate appropriate lines as a positive result (*SI Appendix*, Fig. S1), with the nanoparticle-bound ligands concentrated at the band.

If target DNA was not present in the sample and no amplification had taken place, then only biotin−streptavidin conjugation occurs, which results as a single red line indicating a negative result (*SI Appendix*, Fig. S1). In addition to a control line to show that the lateral flow device worked, the diagnostic device also included a positive control, consisting of a BRCA1 target amplified from artificial DNA targets as lane P, as a quality control step (to rule out false negatives due to test failure in challenging environmental conditions, such as high humidity and heat).

### Laboratory-Based Characterization.

**Fig. 3.**
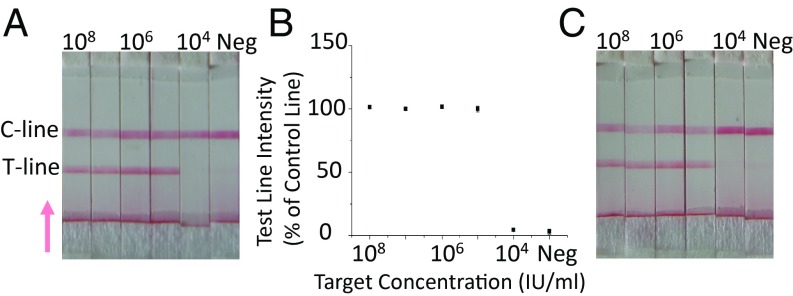
LAMP assay validation. (*A*) The image shows the results of the LAMP-based *Plasmodium* pan assay, which was used to detect several different species of the *Plasmodium* parasite. Each lane represents a paper strip that has been exposed to a sample containing a 10-fold serially diluted concentration of *Plasmodium* standard (30), from 10^8^ IU/mL to 10^4^ IU/mL, together with a negative control (ddH_2_O, Neg). The assay used to detect the different *Plasmodium* species is shown in *SI Appendix*, Fig. S1. The C line represents the control result, and the T line represents the test result. Note that the intensity of the control lines for the negative results are higher than that for the positive results, due to the fact that no beads are captured by the test line. The test line is the first line encountered by the sample as it flows through the lateral flow device (the direction of the flow is represented by the arrow). (*B*) Intensity of the test line shown as a percentage of the control line for each *Plasmodium* (target) concentration level. Data shown are the average of three repeats, and error bars represent the SD. (*C*) Sensitivity of *P. falciparum* LAMP with the 10-fold serially diluted target from 10^8^ IU/mL to 10^4^ IU/mL together with a negative control (ddH_2_O as target). Examples of real-time LAMP curves are available in *SI Appendix*, Fig. S3.

As stated, we first verified the sensitivity of the LAMP assay used in this device in a laboratory setting in the United Kingdom, using a serial dilution of the WHO International Standard for *P. falciparum* DNA (30). Fig. 3*A* shows the test results in the form of the test strips that were generated, together with a quantification of the intensities of the test line to validate the visual analysis (see *Materials and Methods* for details). The analytical sensitivity of the *Plasmodium* pan assay, which detects several *Plasmodium* species (including *P. falciparum*, *P. malariae*, *P. vivax*, and *P. ovale*), was 10^5^ IU/mL after 45-min amplification. The *P. falciparum* assay detected this species (alone) with a similar level of sensitivity as the *Plasmodium* pan assay (Fig. 3*C*), in agreement with previously performed laboratory-based assays, such as commercially available LAMP kits (31).

**Fig. 4.**
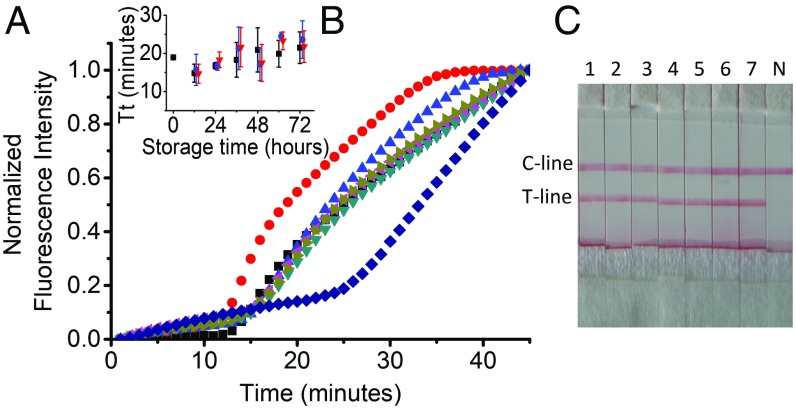
Stability of rehydrated LAMP enzymes. (*A*) Real-time amplification curves of a rehydrated enzyme stored at room temperature for up to 72 h. The real-time amplification curves were normalized to 1 for ease of comparison; 0 h of storage (black square), 12 h (red circle), 24 h (blue triangle), 36 h (dark cyan triangle), 48 h (magenta triangle), 60 h (dark yellow triangle), and 72 h (navy diamond). (*B*) Average threshold time (Tt) for different storage conditions: 4 °C (black square), −20 °C (red triangle), and room temperature (blue circle). Tt was used as a reaction kinetic and represents the time corresponding to 50% of the maximum fluorescence intensity. Data are the average of at least three replicates, and error bars represent the SD. (*C*) Enzyme stability assayed using paper strips; 1, 0 h; 2, 12 h; 3, 24 h; 4, 36 h; 5, 48 h; 6, 60 h; and 7, 72 h of storage at room temperature. N is the negative control.

We also evaluated the performance of rehydrated amplification enzymes after storage at room temperature. Freeze-dried enzymes are commercially available in large batches, but, once rehydrated, their stability becomes a key concern for the application of NAATs in resource-limited areas, since there is limited availability of cold storage facilities (32). We stored rehydrated LAMP mixtures under three different conditions (−20 °C, 4 °C, and at room temperature, i.e., ∼22 ± 2 °C) for 72 h, and measured their activity every 12 h, using a target concentration of 10^5^ IU/mL, by real-time PCR and by using the DNA detection strip used in the microfluidic device (Fig. 4 *A* and *C*). The results demonstrated that rehydrated enzymes stored for 3 d at room temperature showed no significant decrease in performance (Fig. 4*B*), supporting the use of these commercially available kits for field-based trials, where cold storage is unavailable.

We repeated this experiment after the field study in Uganda, using a sample of freeze-dried enzyme that was brought to Uganda, stored there at local room temperature (*ca*. 20 °C to 35 °C) and brought back to the United Kingdom. *SI Appendix*, Fig. S4 shows that the rehydrated LAMP reagent is stable after a day of storage at temperatures up to 40 °C.

#### Clinical Study.

The paper-based microfluidic device reported here was assessed at rural primary schools in Mayuge and Apac Districts in Uganda. Before clinical evaluation, an ethical assessment was carried out with the Ministry of Health in Eastern Uganda, including the treatment of children postdiagnosis. Consent for the testing of children was obtained from the parents, and a census was established to ensure that each child was correctly associated with the parent who had given consent (see *Materials and Methods* for details).

Blood samples from 67 children (aged from 6 y to 14 y old, with equal sex ratios) were tested using the paper-based microfluidic device. The results were compared with microscopy-based diagnosis (performed by a trained and experienced technician from the Vector Control Division of the Ministry of Health, Uganda) and with a malaria RDT (see *Materials and Methods* for details), performed in the field using the same blood sample.

To resolve discrepant results, finger-prick whole-blood samples were also spotted onto Whatman FTA classic cards, which then underwent retrospective, double-blind testing (see *Materials and Methods* for details) by real-time PCR using 18S rRNA gene subunit-based *Plasmodium* sp. screening and *P. falciparum* species-specific screening (6).

The results of the screening are summarized in Table 1 for 59 of the 67 samples (eight samples were used to evaluate a frugal heating method, on a cooking stove, sourced locally, as discussed further). Individual results, with ages and sexes of the children, are available in *SI Appendix* (including individual Ct values; see Dataset S1).

**Table 1.** Diagnostic accuracy of the paper-based microfluidic device, and of microscopy and RDT assays, compared with the diagnosis of *Plasmodium* pan infection by real-time PCR

Field-based diagnostic tests	Real-time PCR		Sensitivity (%)*	Sensitivity CI	Coincidence rate (%)^†^	Coincidence rate CI
	Positive	Negative				
CareStart Lateral Flow Test^‡^						
Positive	46	0	82	77.0–87.0	83	78.1–87.9
Negative	10	3	82	77.0–87.0	83	78.1–87.9
Microscopy						
Positive	48	0	85	80.4–89.6	86	81.5–90.5
Negative	8	3	85	80.4–89.6	86	81.5–90.5
Paper-based microfluidic device						
Positive	55	1	98	96.2–99.8	96	93.4–98.6
Negative	1	2	98	96.2–99.8	96	93.4–98.6

* Defined as number of positives (positive by both the test and real-time PCR) over the total number of positives (by real-time PCR).

^†^ Defined as the number of samples for which results agree between the test and real-time PCR over the total number of samples.

^‡^ The RDT results were counted as being positive if either of the bands for *P. falciparum* or *Plasmodium* pan were positive.

The regions where the tests were carried out are endemic for malaria, and, out of the 67 samples tested, only three were negative for the reference real-time PCR test. The extremely low number of negative samples does not allow the specificity of the device to be assessed. We note that one negative sample tested as being positive by the paper-based microfluidic device. To validate the capability of the device to detect negative samples in a clinical setting, we tested 20 blood samples from healthy patients as a separate study (*SI Appendix*, Fig. S2), validated with PCR. The paper-based tests correctly identified all samples (as the experiments were performed in the laboratory, these results are not included in our analysis of the performance of the devices in the field; Tables 1 and 2).

**Table 2.** Diagnosis of *P. falciparum* infection using the paper-based microfluidic device and RDT, compared with real-time PCR

Field-based diagnostic tests	Real-time PCR		Sensitivity (%)*	Sensitivity CI	Coincidence rate, %^†^	Coincidence rate CI
	Positive	Negative				
CareStart Lateral Flow Test^‡^						
Positive	46	0	82	77.0–87.0	83	78.1−87.9
Negative	10	3	82	77.0–87.0	83	78.1−87.9
Paper-based microfluidic device *P. falciparum*						
Positive	52	1	93	89.7–96.3	92	88.5–95.5
Negative	4	2	93	89.7–96.3	92	88.5–95.5

* Defined as number of positives (positive by both the test and real-time PCR) over the total number of positives (by real-time PCR).

^†^ Defined as the number of samples for which results agree between the test and real-time PCR over the total number of samples.

^‡^ The RDT results were counted as being positive if either of the bands for *P. falciparum* or *Plasmodium* pan were positive.

Taking into account the tests for *Plasmodium* pan and *P. falciparum*, the paper-based microfluidic device had a sensitivity of >98% and a coincidence rate of 97% (95% CI, 91.99 to 100%), compared with the test results generated by real-time PCR (Table 1). Importantly, this sensitivity is above the minimal performance target set out by WHO and FIND (12).

Our method was also more sensitive than the microscopy detection method used (which detected infection in 86% of the tested cases). The performance of both microscopy and RDTs (82% sensitivity) was similar to those obtained by previous studies and was not atypical of what might be expected (10, 27). It is particularly interesting that many RDT tests perform less well in the field (10) than when carried out in more controlled conditions; for example, the CareStart test, used in this study has a sensitivity of 95.4% when performed in a hospital (11).

The paper-based microfluidic device reported here only failed to detect one positive sample, which had a Ct value of 30 on the real-time PCR benchmark assay, and therefore was not a low parasitaemia titer.

We also note that the device can effectively enable the concentration and amplification of an analyte from a relatively large volume of fluid in the finger-prick sample into small volume on the lateral flow strip, a critical challenge for microfluidics. Consequently, healthcare workers are able to differentiate *P. falciparum* infections from other species. Compared with species-specific real-time PCR amplified assay, the sensitivity of the device for diagnosing specific *P. falciparum* infections was 93%, which is better than that of RDTs (82%) in our work, and again equivalent to published results (33).

We also evaluated the capability of the device to be used with minimal power requirements. For eight samples (see rows 66–73 in Dataset S1), we also performed the LAMP reaction on a local cooking stove, using a water pan to heat the device. The temperature of the water was controlled visually using a thermometer (*SI Appendix*, photo ESI-7), varying around 63 °C ± 4 °C. Using this method, the paper origami device correctly detected four positive samples (also positive by PCR) and two negative samples (also negative by PCR). One of these positive samples was negative by both RDT and microscopy. One additional positive sample was not detected by the paper device, but this sample was also not detected by any of the other field-based methods. One sample tested positive by the paper device and negative for all other techniques. These observations would indicate that the paper device, when operated using this frugal heating source, is more sensitive than RDT and microscopy but less sensitive than laboratory-based PCR.

## Discussion

Species-specific multiplexed diagnosis for infectious diseases using either immunodiagnosis or nucleic acid testing is typically performed in centralized laboratories using blood samples processed by trained personnel who require access to specialized instrumentation (34). Although traditional multiplexed RDT lateral flow devices for immunoanalysis exist, their performance is less than needed in the field (10). In our study, we note that the positive predictive values for the immuno-RDTs were very good 46/(46+0 false positive), indicating that, if the diagnostic test was positive, the patient is very likely to have malaria. However, the negative predictive value for the RDT was 3/13, indicating that, if the test gave a negative result, most often it was incorrect.

In an attempt to overcome the problems associated with immuno-RDTs, a new highly multiplexed microfluidic immunodiagnostic platform has recently been deployed in Kenya, although the device requires laboratory-like conditions and further optimization for its performance to be equivalent to reference tests (35). Multiplexed PCR techniques for NAAT diagnosis of infectious disease in resource-limited environments have also been reported, using lateral flow for end point detection (22). Although this has the potential to bring NAAT-based diagnostics closer to patients in rural communities, the assay required conventional equipment, and samples were stored in a field laboratory facility (24).

LAMP isothermal amplification holds significant promise in decreasing the complexity of the instrumentation required for NAAT, although current applications of PCR and LAMP both still remain restricted to well-equipped laboratories and thus are not suitable for low-resource settings, unless field laboratories are established locally (36). To date, the integration of PCR and/or LAMP within very low-cost paper tests, although desirable, has remained challenging, particularly in the context of instrumentation requirements. For example, sample pretreatment is an essential first step for extracting and enriching the DNA of parasites before diagnosis. The subsequent amplification and detection steps also remain highly dependent on temperature control, and low-cost, portable detection techniques are not readily available (37).

Local environmental conditions also present challenges in performing such multiplexed tests as “extreme point-of-need” (38) diagnostics, and these cannot be underestimated. For example, local variations and extremes in temperature and humidity can affect reagent stabilities (39) as well as rates of evaporation and speed of fluid movement during sample introduction into microfluidic matrices. Similarly, care is needed in understanding and controlling contamination between patients’ samples, particularly for NAAT-based technologies being delivered locally, where standard GMP molecular biological processes cannot readily be carried out. In this latter respect, the integration of both internal positive and negative controls within the multiplex is essential to ensure correct diagnosis.

The collection of dried blood spots on FTA cards can, in principle, overcome many of these problems (particularly in urban environments or, for example, in refugee camps). However, as a strategy, this remains impractical in the majority of remote rural settings, where populations are highly dispersed, and where the long transfer times between sample collection, analysis of results, and the point of care makes the delivery of species-specific treatment a significant burden, in terms of both its economics and logistics.

Currently, significant advances are occurring within the field of paper microfluidics and have recently been extensively reviewed (21, 40, 41). For example, Connelly et al. (7) developed a “paper machine” for molecular diagnosis of *Escherichia coli* from human plasma using a magnetic device. To our knowledge, the use of “origami” devices has previously been either for immunoassays [as ELISAs (42)] or only as a preanalytical self-contained device for sample preparation, with no analytical process integrated (43).

Here we combine sample processing from whole blood together with an easy-to-read visualization for the rapid readout of diagnostic information. The simple manufacturing techniques, which include hot wax printing and laser cutting, enabled devices to be assembled within the laboratories at a cost of ∼$0.50 per assay (triplex assay cost is <$2.00). The cost could be further reduced in the future if manufactured at scale or in an industrial setting. Device reliability during manufacture was extremely good, and a robust quality control protocol ensured that there were no failures in the field.

## Conclusions

Our results demonstrate that paper-based microfluidic devices can deliver precision diagnostics for malaria in low-resource, underserved settings with a sensitivity that is higher than that of the current malaria diagnostic tests used in the field (such as microscopy and RDTs) and with performance that is similar to that of a laboratory-based real-time PCR test. These diagnostic devices could have a meaningful, positive impact on the provision of mass screening and treatment in campaigns to eliminate infectious disease (14). These campaigns have had limited success to date in combating malaria transmission, which has been linked to the inability of current field-based diagnostic tools to detect low-level infections (44). Thus, the availability of easy-to-use, highly sensitive NAATs, such as those provided by this device, could potentially detect these missed cases and reduce the opportunity for transmission. This not only would have a significant impact on public health in areas where malaria is highly prevalent, but could also inform current thinking within governments and nongovernmental organizations concerning improvements in the effectiveness and cost-effectiveness of prophylactic approaches to control diseases (where new precise diagnostic tools are required to rapidly and accurately target where treatment is needed).

## Materials and Methods

### Materials and Reagents.

Deionized water (DI H_2_O) with a resistivity of >18 MΩ·cm was used to prepare all aqueous solutions transported from the United Kingdom. Fresh bottled drinking water was purchased locally and used for washing glassware and, for example, preparing Giemsa stains. Unless otherwise specified, all other reagents were purchased from Sigma-Aldrich.

### Multiplex LAMP System.

The primer sets used for the LAMP assay were based on previously published primer sequences for *P. falciparum* (45), and *Plasmodium* genus (31). A LAMP assay that amplified a BCRA1 gene fragment served as a positive control (46). The primers were all purchased from Eurofins Genomics. The sequences are provided in *SI Appendix*, Table S1. The 20-µL LAMP reaction contained 0.5 µM inner primers (forward inner primer and backward inner primer), 0.1 µM outer primers (F3 and B3), and 0.4 µM loop primers, labeled with Biotin and FITC at their 5′ ends. The reaction mix also contained 0.4 mM of each dNTPs (Sigma), 4.0 mM MgSO_4_, 50 mM Tris⋅HCl (pH 8.1), 30 mM KCl, 30 mM (NH_4_)_2_SO_4_, 0.1% Triton X-100, 1 M Betaine, and eight units of Gsp SSD 2.0 DNA polymerase (freeze-dried; OptiGene). The multiplex LAMP reactions were performed at 65 °C for 45 min.

### Paper-Based Microfluidic Device.

The fabrication of the device was performed, without specialized facilities or a clean room, simply by using a wax printer (Xerox ColorQube 8570) (27, 47) and a hot plate. Each device contained a filter paper-based fluidic device where the extracted DNA and the sample liquid were constrained by printed hydrophobic wax (cat# 108R00935; Xerox).

The filter paper was first printed with the wax using the wax printer, then heated at 120 °C for 1 min to 1.5 min on the hot plate to melt the printed wax. The melted wax diffused through the filter paper, thus forming the same hydrophobic pattern of channels on both sides. Subsequently, the glass fiber spots (3 mm in diameter; Whatman) were manually positioned, such that, when the paper was appropriately folded, the reagents and extracted DNA could be transferred to the spots. After adding the LAMP master mix, the plastic device was sealed using an acetate film, preventing liquid evaporation during amplification.

### Lateral Flow Test.

The plastic devices containing finger pumps and lateral flow strips (Fig. 1) for field testing were manufactured by CNC machining at Epigem Ltd. The dimensions are provided in *SI Appendix*, Fig. S5. A single-sided adhesive acetate film (MicroAmp Optical Adhesive Film; Thermo Scientific) sealed the plastic device plate with the LAMP reaction chambers, which contained glass fiber spots that received DNA from the paper fluidic device.

### Quality Control.

Air bubbles, generated during assembly of the paper strips onto the plastic case, can lead to critical failure during processing. All devices were inspected carefully for such air gaps and discarded if gaps were present. This process was repeated before each assay. As stated, the devices’ designs were fully characterized in a laboratory setting before field use, with no subsequent additional quality check performed in the field.

As stated, we stored rehydrated LAMP mixtures under three different conditions (−20 °C, 4 °C, and at room temperature, i.e., ∼22 °C) and checked their stability both by PCR and by using the DNA detection strips, to show that rehydrated enzymes can be stored for 3 d at room temperature (as cold storage was not available). The use of both a positive control for the LAMP reaction and a control line in the lateral flow assay enabled the validation of results in the field.

### Study Design.

The aim of this study was to demonstrate the performance of an origami-lateral flow format, combining simple sample processing with easy visualization of results. One additional objective was also to demonstrate the potential role of multiplexed species-specific diagnosis from whole blood finger pricks in rural, underserved communities. The diagnostic strips were used to test blood samples collected from 67 school children from Bukagabo Primary School, in the St. Kizito region, and Mayuge and Apac District School, Apac region. The sample size was calculated to provide enough samples for a clear evaluation of sensitivity (with a power of >0.8 and *P* value of <0.05 for a target sensitivity of >90%, yielding >30 positive samples), while, at the same time, taking into account the practical limitation of carrying out the test (limiting the numbers to 10/d to 20/d). Given the high prevalence (expected above 70% based on epidemiological data, but, in practice, often >90% in our study), we were not able to test for specificity (48).

This study was conducted with the Vector Control Division of the Ministry of Health in Kampala, Uganda, on neglected tropical diseases, and was approved by the Vector Control Division Research and Ethics Committee, VCDREC/078, and Uganda National Council for Science and Technology (HS 2193). School children between the ages of 6 y and 14 y were recruited as a cohort (balanced for sex) under the supervision of their class teachers. Each child was assigned a unique identification number (ID), and their age, sex, name, parents’ name (to ascertain consent), and school class were recorded. Details without names were computerized and anonymized using the ID numbers. No personal data were revealed to the investigators. Written informed consent of the parents and the head teacher were also obtained.

The parents/guardians were informed of the activities by the head teacher at community meetings, and their verbal and written assent for their children’s participation was obtained. Where parents were unable to write, their fingerprint was used as a signature (see *SI Appendix*. photos ESI-1 and ESI-2). After testing, all patients were treated as malaria cases and given antimalarial therapeutics, including cases that tested negative on site. This follows guidance in the ethical approval obtained, given that all samples were retested by PCR in the United Kingdom retrospectively, a method that is more sensitive than the ones available onsite.

Ministry of Health Vector Control Division healthcare workers collected all blood samples as finger-prick samples (<50 µL) either in 1.5-mL Eppendorf tubes [for field reference methods, i.e., for Giemsa staining (see *SI Appendix*, photo ESI-3) and microscopy, for CareStart Malaria RDTs (see *SI Appendix*, photo ESI-4), or for storage on Whatman FTA (see *SI Appendix*, photo ESI-5) classic cards and retrospective PCR analysis in the United Kingdom] or for being placed on the paper device; see *Microfluidic Testing* for all details. As stated, all samples were anonymized with an ID. Microfluidic testing, Giemsa staining, and microscopy as well as diagnostic assessment with the RDT were all carried out within the same school classrooms in parallel to testing on the paper-based microfluidic device without sample storage. Testing was double-blinded between on-site and reference tests. After testing at the school, all used paper devices and small plastic consumables were incinerated by burning on a bonfire, while glass slides and RDTs were stored in a biohazard container for safe disposal at the Vector Control Division, Kampala (*SI Appendix*, photo ESI-6).

We also provide data from a separate study of 20 negative samples, in *SI Appendix*, Fig. S2, obtained in September 2018 from Adicon clinical laboratories (Hangzhou, China) and validated on PCR. Five milliliters of whole blood was obtained from patients using routine phlebotomy in a vacutainer, coated with heparin. The blood was stored in a fridge until use (for less than 8 h). Five microliters of the sample was taken out of the tube and used on the paper device, while 200 µL was processed for PCR (see *Real-Time PCR*). This latter work was performed in a laboratory and in accordance with the ethical standards of the institutional and national research committee and with the 1964 Helsinki declaration and its later amendments or comparable ethical standards. All procedures were carried out in accordance with *Measures on Ethical Review over Biomedical Research Involving Human Subjects* (49). All of the samples were collected from patients after obtaining informed consent, and the data from these patients were used in this study only.

### Microfluidic Testing.

In practice, three assays were typically run in parallel as a triplex, which required ∼45 min to complete. All analysis was performed in the children’s classrooms. Five microliters of human whole blood was pretreated with 1 µL of proteinase K (20 mg/mL; Thermo) and 10 µL of lysis buffer (4 M GUSCN, 25 mM sodium citrate, 0.2% SDS) in a 1.5-mL Eppendorf tube and incubated at 60 °C for 5 min with 10 µL of cell lysis buffer, and 1 μL of MagaZorb beads (diameter 4.5 μm) (Promega). Twenty microliters of binding buffer (from MagaZorb kit; Promega) was mixed with the sample and incubated at room temperature for 5 min. A 40-µL volume of the resulting homogenous mixture was then added to the sample zone (Fig. 2*B*). The beads remained at the surface of the sample zone while residues were washed away to the waste pad through the filter paper.

The magnetic beads were washed with volume-independent washing buffer (from MagaZorb kit; Promega) twice. The device was then folded to bring the sample zone into contact with the distributing channel, before 40 µL of elution buffer (100 mM TE buffer, pH 8.0) was added to elute the DNA. The eluted DNA was then guided to three different locations on the paper within the wax-printed channel and transferred by capillary action to their respective reaction chambers in the plastic device by making contact with a paper pad inserted into the plastic chamber. The folded paper was then torn away and incinerated for safe disposal. The independent chambers were sealed with an acetate film to prevent liquid evaporation during the isothermal amplification, following the addition of 16 µL of species-specific LAMP reagents.

The LAMP isothermal reaction (*n* = 59) was performed on an electric-powered hot plate, although, to demonstrate that the assay could be performed without electric power, a small number (*n* = 8) were amplified in a water bath, using a pan on a local cooking stove. The temperature of the assays was maintained at around 63 °C by racking the chips in a metal block with a high thermal heat capacity (sitting in water) and, when necessary, controlling the distance between the pan and stove (see *SI Appendix*, photo ESI-7). Temperature variations were ±3 °C around the target temperature. As previously stated, and in all cases (*n* = 67), at the end of the incubation, the running buffer chambers (1 in Fig. 1) were manually pressed to enable the buffer to push the LAMP reaction mix onto the lateral flow device, as previously published (28), manufactured by Ustar Biotech Co., Ltd. Microfluidic DNA test strips were imaged using an iPhone 6 camera on automatic setting, as a digital record. No postprocessing was required for line intensity analysis and diagnosis. *SI Appendix*, photo ESI-8 shows an example of such a strip.

Notwithstanding this, the intensities of the test and control lines were analyzed and measured using ImageJ (50), and the intensity of the control line (in the red channel) was divided by the intensity of the test line for each strip to obtain the percentage intensity of the control line for each test (51).

### Thick Blood Smears, Giemsa Staining, and Microscopy.

Optical microscopy was performed by trained technicians working for the Vector Control Division of the Ministry of Health, Kampala. All healthcare workers were qualified and all had >2 y of experience of field microscopy. *SI Appendix*, photo ESI-3 shows Giemsa staining and microscopy, with testing performed in the school classrooms at the same time as microfluidic testing and RDT analysis. For the thick film stain, a drop of blood from the finger prick was placed at the center of a glass slide and spread as a smear. The slide was allowed to dry under ambient conditions for 20 min and was then stained with 10% Giemsa stain for a further 10 min.

Smears were examined under a compound microscope (made in Philippines; Model CX21FS1; Olympus Corporation) using a 100× oil immersion objective lens, with either electrical or natural light sources (depending upon availability). Malaria parasites were counted against 200 white blood cells (WBCs) and multiplied by factor 40 to give a parasitaemia per microliter of blood (in 1 µL of blood) (52).

### RDT Analysis.

As stated, CareStart Malaria RDTs, provided by the Ministry of Health, were used as a field reference technique, performed in the school classrooms (see *SI Appendix*, photo ESI-4) at the same time as microfluidic testing and thick film smears. We used the One step Malaria HRP-II (*P. falciparum*) and pLDH (*P. pan*) Antigen Rapid Test (SD Bioline Malaria Ag) *P. falciparum*/*Plasmodium* pan test kit as a qualitative test method of detecting species-specific *Plasmodium* of *P. falciparum* and *Plasmodium* pan (*P. vivax*, *P. malariae*, or *P. ovale*) antigens in the individuals’ blood.

A finger prick of blood (5 µL) was transferred from the child’s finger to the circular sample well. Four drops of CareStart assay diluent were then added into the square assay diluent well, and the test device was allowed to stand for 20 min (for capillary flows to enable the immunodiagnostic binding). A positive *P. falciparum* was indicated by the presence of two colored bands (“*P. falciparum*” test line and “C” control line) or three colored bands (“*P. falciparum*” and “*Plasmodium* pan” test lines and “C” control line) within the result window. Positive for other *Plasmodium* species (*P. vivax*, *P. malariae*, or *P. ovale*) was indicated by the presence of two colored bands (“pan” test line and “C” control line) within the result window regardless of which band appears first. Mixed infections of *P. falciparum* and *P. vivax* (or *P. malariae* and *P. ovale*) were indicated by the presence of three colored bands (“*P. falciparum*” and “*Plasmodium* pan” test lines and “C” control line) within the result window regardless of which band appears first. No malarial infection was indicated by one line “C” in the result window [2015 One Step Malaria HRP-II (*P. falciparum*) and pLDH (pan) Antigen Rapid Test “SD BIOLINE Malaria Ag P.f/Pan test kit”; Standard Diagnostics, Inc.; https://www.alere.com/en/home/products-services/brands/sd-bioline.html].

### Dried Blood DNA Extraction.

Finger-prick whole-blood samples were spotted on FTA classic cards (Whatman) directly after bleeding, air-dried completely, and, on return to the Vector Control Division, Kampala, shipped with a desiccant at ambient temperature. Upon arrival in the United Kingdom, they were stored at −20 °C until used. Three-millimeter sample discs from the dried sample spot were removed using a disposable punch and placed in a clean tube. Three hundred microliters of PBS was added to the tube, which was then incubated at room temperature for 2 h, after which time 20 μL of protein K was added, and the sample was incubated at 56 °C for 1 h. The samples were further processed following the instructions from the MagaZorb DNA Mini-Prep Kit (Promega) and eluted with 50 μL of elution buffer. The extracted samples were coded randomly and dispensed in a 96-well plate together with 10 negative (water) samples added at random.

### Real-Time PCR.

The 18S rRNA gene subunit-based *Plasmodium* sp. screening real-time PCR and *P. falciparum* species-specific real-time PCR provided the reference standard for assessing the diagnostic performance of the paper-based microfluidic device reported in this program of work (6). The reaction was performed as a double-blind study, in a final volume of 25 μL, which contained 5 μL of DNA, 10 μL of Brilliant multiplex QPCR Master Mix (Agilent Technologies), 200 nM forward primer, Plasmo1 (5′-GTTAAGGGAGTGAAGACGATCAGA-3′), and 200 nM reverse primer, Plasmo 2 (5′-AACCCAAAGACTTTGATTTCTCATAA-3′). The TaqMan probe, Plasprobe: 5′-FAM-ACCGTCGTAA TCTTAACCATAAACTATGCCGACTAG-TAMRA-3′ and Falcprobe: 5′-FAM-AGCAATCTAA AAGTCACCTC GAAAGATGACT-TAMRA-3′ were used. The real-time PCR was performed in an ABI 7500 fast real-time PCR instrument (Applied Biosystems) with the following thermal profile: 95 °C for 10 min, and 45 cycles of 95 °C for 15 s and 60 °C for 1 min. The sample was considered positive by identifying the threshold cycle number (Ct) at which normalized reporter dye emission raised above background noise. If the fluorescent signal did not increase within 40 cycles (Ct 40), the sample was considered negative.

The PCR was run on double-blinded samples by a different technician than the one who had performed the DNA extraction. The results were recorded by a third person and aligned with the blinded samples. Ten water samples were added at random in the plate and were all negative. Two positive samples showed a Ct value between 39 and 40. These samples were retested (reextracted), and the final Ct was found to be 26 and 38. Individual results are available in *Dataset S1*.

### Statistics and Analysis (Including PCR).

As stated, the intensity of the test and control lines was analyzed and measured by using ImageJ (50). The intensity of the control line (in the red channel) was divided by the intensity of the test line for each strip to obtain the percentage intensity of the control line for each test (51).

Confidence intervals for sensitivity data (Tables 1 and 2) were calculated on the basis of the binomial distribution using the STATA 14.1 statistical package (StataCorp LP).

### Data and Materials Availability.

All data are available open access in the University of Glasgow repository (53).

## Supplementary Material

Supplementary File

Supplementary File

Supplementary File
