# Supplementary material for: Paper-based microfluidics for DNA diagnostics of malaria in low resource underserved rural communities
Source: Proc Natl Acad Sci U S A. 2019 Feb 19;116(11):4834–42. doi: 10.1073/pnas.1812296116 (PMC6421471; doi:10.1073/pnas.1812296116)
Supplement: Supplementary File [file pnas.1812296116.sapp.pdf]

PNAS
www.pnas.org

Supplementary Information for

Paper-based Microfluidics for Diagnosing Malaria in Low Resource Rural Environments

Julien Reboud, Gaolian Xu, Alice Garrett, Moses Adriko, Zhugen Yang, Edridah M. Tukahebwa, Candia Rowell, Jonathan M. Cooper

Jonathan M. Cooper
Email: Jon.Cooper@glasgow.ac.uk

This PDF file includes:

Figs. S1 to S5
Table S1
Caption for movie M1
Caption for data file S1
Photo ESI 1-7

Other supplementary materials for this manuscript include the following:

Movies M1
Dataset S1

www.pnas.org/cgi/doi/10.1073/pnas.1812296116

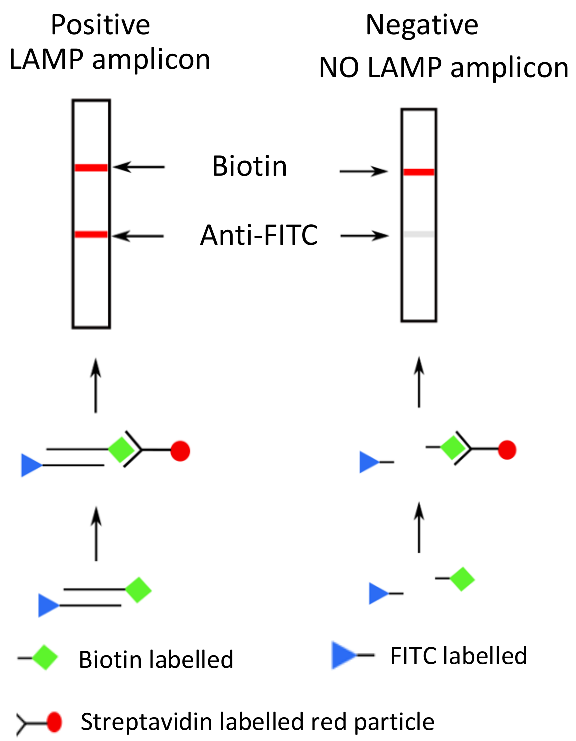

Fig. S1. Principle of the lateral-flow strip for DNA detection. Only the biotin and FITC dual-labelled amplicon can be detected at the test line, forming an intense red line, as the beads become trapped in a defined area. The biotin deposited on the strips serves to trap the streptavidin-labelled red beads which are not linked to a FITC-primer, showing that the lateral flow strip is working correctly.

®

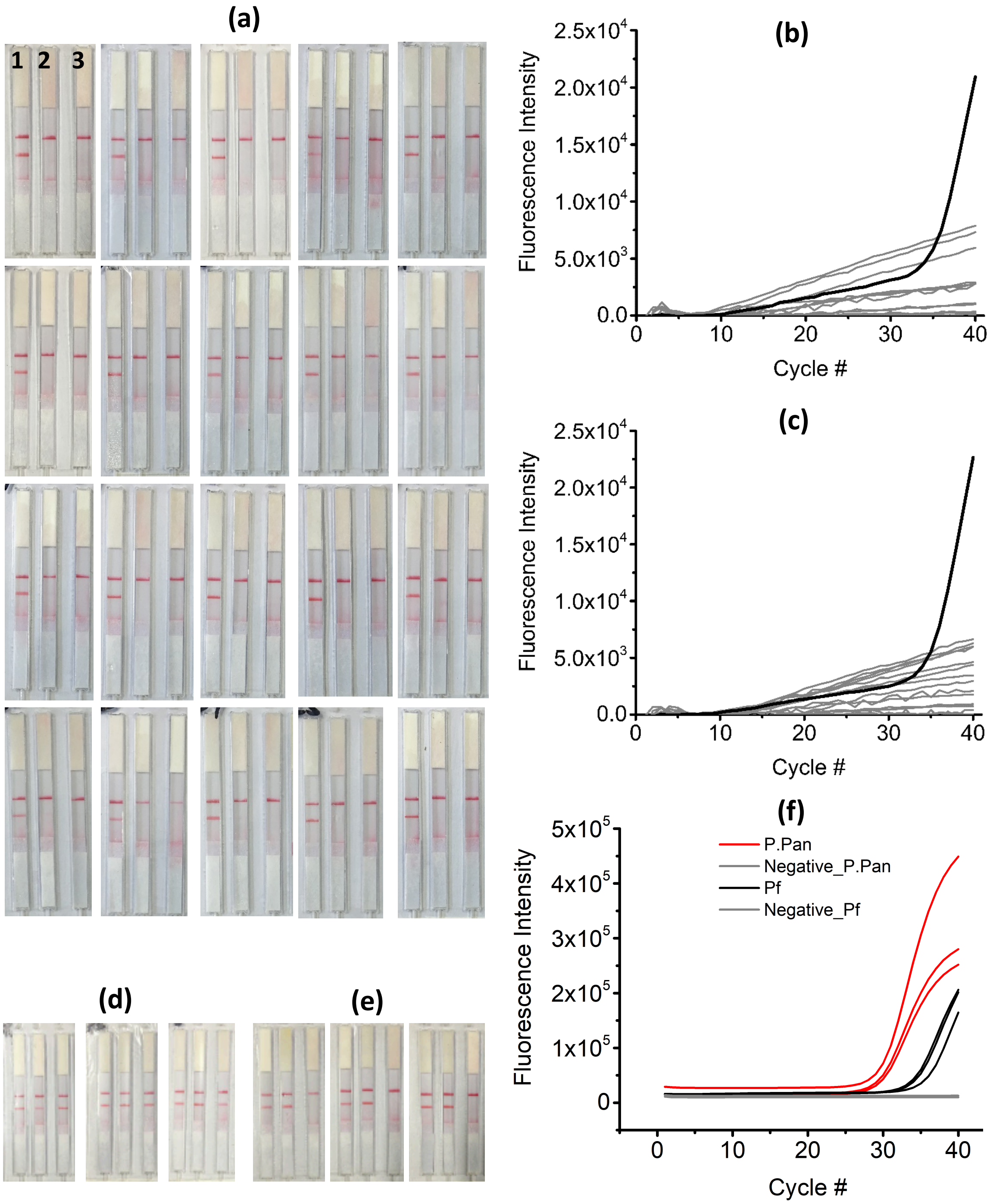

Fig. S2. (a) 20 negative (healthy patients) samples were tested on the paper origami device in a separate study. Line 1 – positive control based on the tuour-suppressor BRCA1 gene; Line 2 *P.Pan*,; Line 3 *P.f.* (b-c) PCR curves for *P.pan* (b) and *Pf* (c). Note that the positive controls come off the background at ca. cycle 34. Samples were also spiked with amplicons for *P.Pan* and *Pf* (d) and *P.Pan* only (e) to confirm the reactions, validated by PCR (f). The amount of the DNA spiked in the samples was 1000 copies.

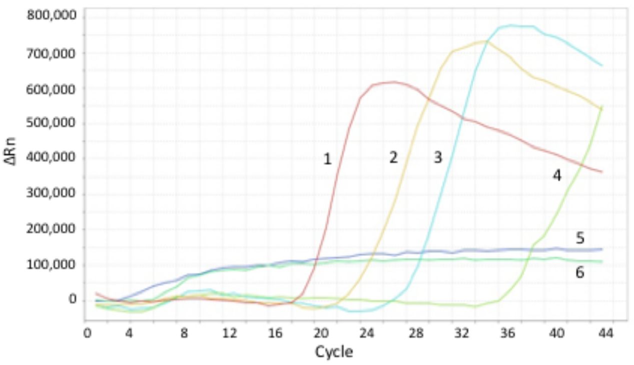

Fig. S3. Real-time amplification curve of P. falciparum LAMP with the 10-fold serially diluted target (1- 5), and ddH_2O as a negative control (6): 1. 10^8 IU/mL; 2. 10^7 IU/mL; 3. 10^6 IU/mL; 4. 10^5 IU/mL; 5. 10^4 IU/mL; 6. Negative control (no target DNA).

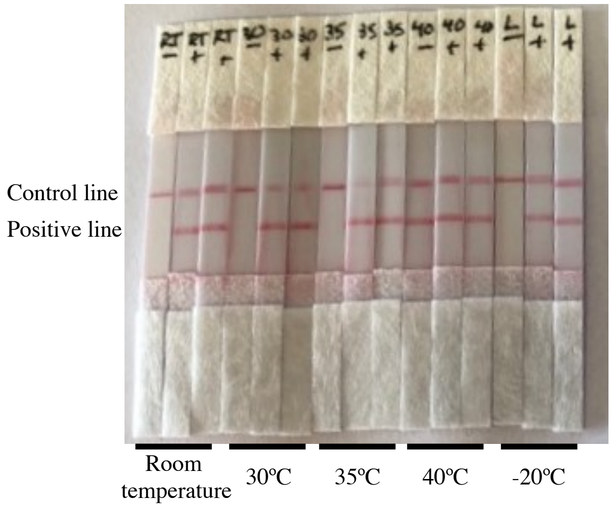

Fig. S4. Reagent stability. A bottle of freeze-dried enzyme that was carried to Uganda, stored there for 10 days at room temperature and brought back to the UK, was rehydrated as per the manufacturer's recommendations and stored at different temperatures for 24h. LAMP reactions with 5 µl of 10^{-5} IU/ml of DNA were carried out on the qPCR machine. The results were read with the lateral flow strips. For each temperature, there was one negative control (no target) and a duplicate. (L) refers to the positive control where the mix is stored at -20°C.

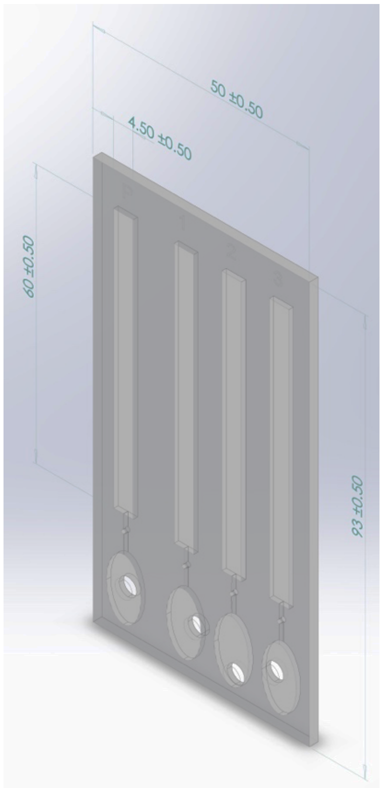

Fig. S5. Dimensions of the plastic cartridge. Measurements are provided in mm with a manufacturing tolerance of 0.5mm. The device is composed of 2 machined plastic sheets (2mm thick), glued together.

Table S1. Primer sequences for multiplex-LAMP reactions

Species	Oligos	Sequence (5’-3’)
Plasmodium-pan	PANF3	5’-TCGCTTCTAACGGTGAAC
	PANB3	5’-AATTGATAGTATCAGCTATCCATAG
	PANLPF5b	5’-Biotin-TGGACGTAACCTCCAGGC
	PANLPR5F	5’-FITC-CACTATACCTTACCAATCTATTTGAACTTG
	PANFIP	5’-GGTGGAACACATTGTTTCATTTGATCTCATTCCAATGGAACCTTG
	PANBIP	5’-GTTTGCTTCTAACATTCCACTTGCCCGTTTTGACCGGTCATT
P. falciparum	PFF3	5’-TGTAATTGGAATGATAGGAATTTA
	PFB3	5’-GAAAACCTTATTTTGAACAAAGC
	PFLPF5B	5’-Biotin-GCACCAGACTTGCCCT
	PFLPR5F	5’-FITC-TTGAATATTAAAGAA
	PFFIP	5’-AGCTGGAATTACCGCGGCTG GGTTCCTAGAGAAACAATTGG
	PFBIP	5’-<br>TGTTGCAGTTAAAACGTTCGTAGCCCAAACCAGTTTAAATGAAAC
BRCA1	BRCA1F3	5’-TCCTTGAACTTTGGTCTCC
	BRCA1B3	5’-CAGTTCATAAAGGAATTGATAGC
	BRCA1LPF5B	5’-Biotin-AGAACCAGAGGCCAGGCG
	BRCA1LPR5F	5’-FITC-GCAGATAGGCTTAGACTCAA
	BRCA1FIP	5’-ATCCCCAGTCTGTGAAATTGGGCAAAATGCTGGGATTATAGATGT
	BRCA1BIP	5’-GCAGCAGAAAGATTATTAACTTGGG-<br>CAGTTGGTAAGTAAATGGAAGA

Movie M1 illustrates the complete process of the assay using mock colored water to show fluid movement in the device.

Additional data table S1 (separate file)

Datafile.csv This file provides the Ct values from the real-time PCR results after unblinding.

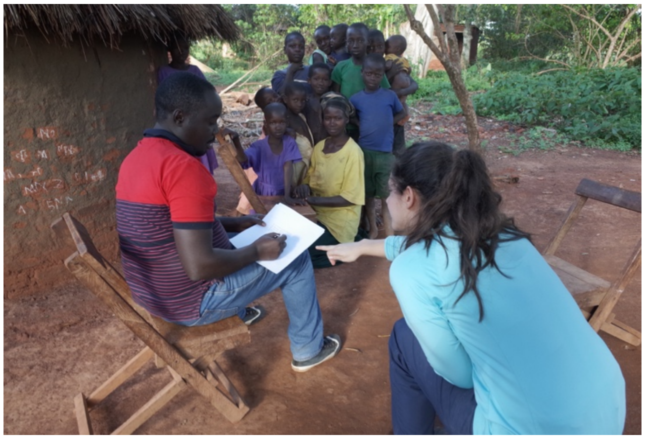

Photo ESI-1 census collection data, recording names of families, addresses and children's ages;

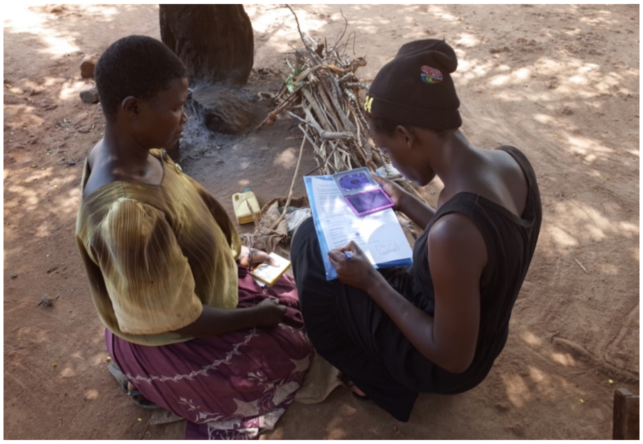

Photo ESI-2 subsequently collecting consent for diagnostic testing using fingerprint signatures from the parent.

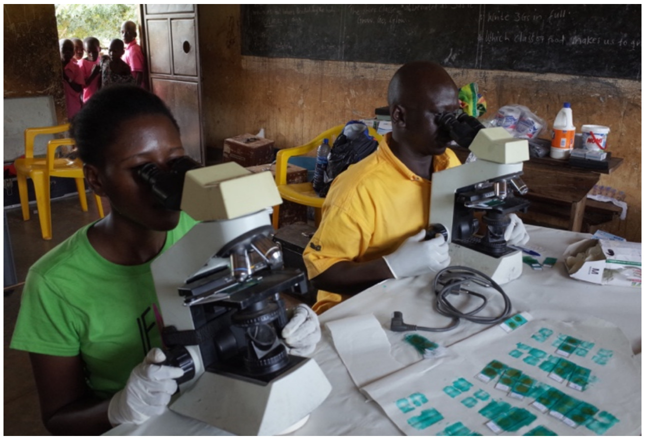

Photo ESI-3 Giemsa staining and light microscopy as a reference technique

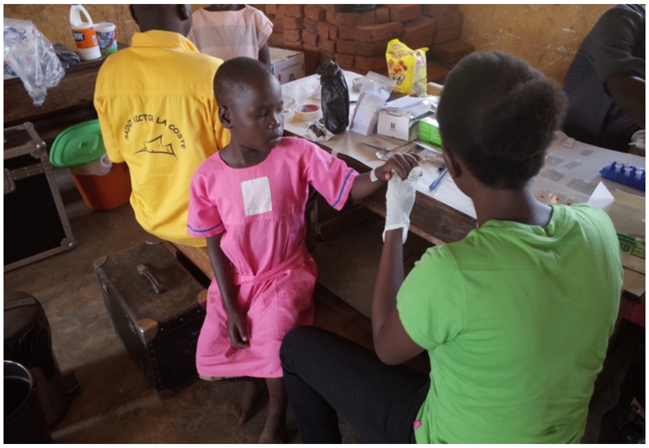

Photo ESI-4 Collecting finger-prick volumes of blood

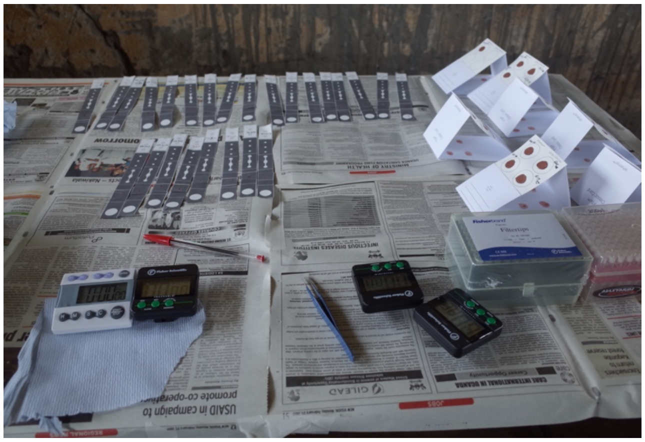

Photo ESI-5 Origami tests for testing in the school with and FTA blood-spots on cards for retrospective analysis using PCR at the University of Glasgow

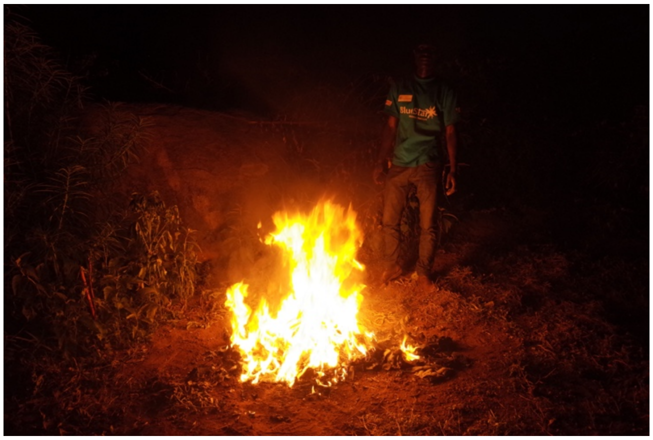

Photo ESI-6 Incineration of paper devices and readily combustible waste at the end of the testing.

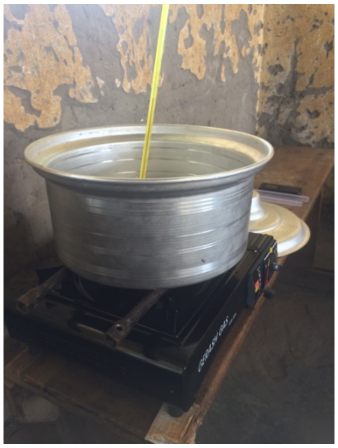

Photo ESI-7 Frugal testing using a local cooking stove to run LAMP amplification using manual control of temperature.
